# Geometric and singularities insights of swept surfaces via the Bishop frame in Euclidean 3-Space

**DOI:** 10.1371/journal.pone.0340461

**Published:** 2026-01-22

**Authors:** Fatemah Mofarreh, Rashad Abdel-Baky, Munirah Alsahli

**Affiliations:** 1 Mathematical Science Department, Faculty of Science, Princess Nourah Bint Abdulrahman, Riyadh, Saudi Arabia; 2 Department of Mathematics, Faculty of Science, University of Assiut, Assiut, Egypt; Southwest University of Science and Technology, CHINA

## Abstract

This study investigates the geometry and singular behavior of swept surfaces generated by the move of Bishop frame along a spatial curve in Euclidean 3-space. The surface is defined as the envelope of a family of unit spheres whose centers trace an axial trajectory, with the contact points forming great circles within a prescribed plane. A parametric representation is established to highlight the dependence of the surface on both the axial and profile curves. Key geometric features including the coefficients of the first fundamental form, unit normal vectors, and curvature characteristics are analyzed, revealing that the profile curves act as planar geodesics and curvature lines. We further examine singularities, offset surfaces, and parabolic curves, deriving conditions for smoothness, geodesicity, and convexity. Special attention is given to the criteria under which the surface becomes developable, particularly when it reduces to a cylinder, cone, or tangent surface. Several illustrative examples, including those arising from mate curves of slant helices and circular trajectories, demonstrate the resulting geometric phenomena.

## 1 Introduction

Channel surfaces, also referred to as tubular surfaces, are generated as the envelopes of a continuously evolving family of spheres. In this context, the centers of the spheres trace a spatial curve known as the spine or axial curve. When the radius remains constant as the sphere moves along this path, the resulting surface constitutes a particular case of a swept surface. More generally, a swept surface arises when a fixed shape such as a circle, curve, or sphere is transported along a prescribed three dimensional trajectory. The overall geometry is primarily governed by the path of the moving shape’s center, while variations in shape introduce finer details to the surface structure. These geometric constructs are not only foundational in theoretical mathematics but also play a critical role in industry, innovation, and infrastructure. They are widely employed in CAD, manufacturing, and architectural modeling. Familiar examples include right circular cylinders, formed when the spine is a straight line with a constant radius; tori, where the spine traces a circular path; and circular cones, in which the spine is linear but the radius varies. Another fundamental type is the surface of revolution, generated by rotating a curve around a fixed axis to create a symmetric shape. These methods extend classical techniques of constructing geometric forms through the rotation and translation of curves and solids [1–8].

Swept surfaces are central to the field of Computer-Aided Geometric Design (CAGD) and have broad applications. They are extensively used in robotic trajectory planning, the creation of blending or transition surfaces (as found in piping systems), and the fabrication of sculpted surfaces in manufacturing [9,10]. Of particular importance is the subclass of developable ruled surfaces, which hold both theoretical and practical significance. This group includes cylinders, cones, and tangent surfaces forms that are essential in motion analysis, vehicle engineering, and marine structure design [11–16].

The foundational concept of the Bishop frame was first introduced by Bishop (1975) as a rotation minimizing alternative to the classical Frenet frame, later refined for sweeping and motion planning applications by Klok [17] and the computational formulations of Wang and others (1997, 2008), which established its stability and efficiency in Computer-Aided Geometric Design [12,18]. Building upon these geometric frameworks, Deshmukh et al. (2018) extended the analysis to the theory of natural mate curves in Euclidean 3-space, forming a direct bridge between curve theory and the differential geometry of swept surfaces [17]. Abdel-Baky has contributed to a series of works focusing on sweeping surfaces in various geometrical settings using adapted frames such as the Bishop frame, Darboux frame, etc. He studies sweeping surfaces generated by the natural mate of a spatial curve in Euclidean 3-space under different considerations such as using Bishop frame, developing a Bishop frame for the conjugate or curve in a 3-dimensional Lie group [19–22]. The natural mate of a space curve is defined such that its tangent aligns with a principal normal of the original curve at each point. It is important because it is providing an alternative curve with interesting differential geometric properties. At [19], Al-Jedani and Abdel-Baky introduce a new Bishop frame built along the natural mate of a given space curve, then they use that frame to generate a sweeping surface whose parametric curves are curvature lines, and they derive necessary and sufficient conditions for this sweeping surface to be a developable ruled surface. Thus, the concept of natural mate plays the role of the spine curve along which the frame is built and the sweeping is done.

This study examines the geometric structure and singular behavior of swept surfaces generated via the Bishop frame along a spatial curve in Euclidean 3-space. These surfaces arise as the envelopes of a one parameter family of unit spheres whose centers follow a prescribed axial trajectory, with points of contact forming great circles within a fixed plane. A parametric representation is developed to explicitly capture the dependence of the surface on both the axial and profile curves. Key differential geometric properties including the coefficients of the first fundamental form, unit normal vectors, and curvature measures are systematically derived. It is shown that the profile curves constitute planar geodesics and lines of curvature. The analysis further extends to singularities, offset surfaces, and parabolic curves, with precise conditions established for smoothness, geodesic nature, and convexity. Special attention is given to the developability of the surface, identifying the necessary and sufficient conditions under which the surface reduces to classical forms such as cylinders, cones, and tangent surfaces.

This paper marks a conceptual shift in Professor Rashad Abdel-Baky’s research on Bishop-type swept surfaces from classical developability analysis to a unified geometric investigation of curvature, singularities, and convexity. Whereas his earlier works, particularly with Al-Jedani (2023), generated surfaces via type-2 and type-3 Bishop frames along the natural mate curve, focusing mainly on developable conditions such as cylinders, cones, and tangent surfaces, the present study redefines the framework by adopting the standard Bishop frame along the axial curve itself. The surface is constructed as the envelope of a one parameter family of moving unit spheres, introducing a new geometric mechanism based on tangency rather than translation. This approach expands the analysis beyond developability to encompass singularity characterization, the instantaneous axis of rotation, and precise convexity and parabolic curve conditions distinguishing elliptic and hyperbolic regions. The natural mate here is no longer the generating curve but a theoretical tool linking the Frenet and Bishop frames to describe local geometric transitions. In essence, the paper establishes a deeper structural understanding of how rotation minimizing frames govern the shape behavior of swept surfaces, unifying both axial and mate based constructions within a coherent Euclidean setting.

## 2 Preliminaries

Let ${𝔼}^{3}$ represent Euclidean 3-space [1–9]. Consider a spatial curve γ=γ(u) parameterized by arc length *u*. To ensure the curve is not a straight line, lets assume $\gamma {\;}^{\prime }(u)\neq 0$ for all 0≤u≤L. The Serret Frenet frame (SFF) associated with γ(u) is expressed as $\left\{e_{1}(u\right)$, $e_{2}(u)$, $e_{3}(u)$, κ(u), τ(u)}, where κ(u) and τ(u) denote the curvature and torsion, respectively. The arc length differentiation of this frame follows the equation:

$$\left(\begin{array}{c} e_{1}{\;}^{\prime }\\ e_{2}{\;}^{\prime }\\ e_{3}{\;}^{\prime } \end{array}\right)=\left(\begin{array}{ccc} 0 & \kappa & 0\\ -\kappa & 0 & \tau \\ 0 & -\tau & 0 \end{array}\right)\left(\begin{array}{c} e_{1}\\ e_{2}\\ e_{3} \end{array}\right)=\omega \times \left(\begin{array}{c} e_{1}\\ e_{2}\\ e_{3} \end{array}\right);{\;}^{\prime }=(\frac{d}{du}),$$ (2.1)

where $\omega (u)=\tau (u)e_{1}+\kappa (u)e_{3}$ is the angular velocity of the SFF on α(u).

Although the SFF is relatively simple to compute, its rotation around the tangent of a general space curve can introduce unwanted twisting, making it unsuitable for motion planning and swept surface modeling. Furthermore, the SFF is not continuously defined for a C^1^ -continuous curve, and even for a C^2^-continuous curve, it becomes undefined at inflection points where the curvature κ(u)=0. This discontinuity presents challenges in surface modeling applications [1–4]. To overcome these issues, an alternative frame is constructed along a unit speed curve γ(u). The parallel transport of an orthonormal frame along a curve is achieved by independently moving each component without introducing additional rotation [8]. This approach utilizes the tangent vector along with a suitably chosen basis for the remaining frame elements (see [10] for further details). The resulting frame, known as the rotation minimizing frame (RMF) or Bishop frame (BF), is given by [1,2]:

$$\left(\begin{array}{c} e_{1}\\ e_{2}\\ e_{3} \end{array}\right)=\left(\begin{array}{ccc} 1 & 0 & 0\\ 0 & \cos\vartheta & \sin\vartheta \\ 0 & -\sin\vartheta & \cos\vartheta \end{array}\right)\left(\begin{array}{c} j_{1}\\ j_{2}\\ j_{3} \end{array}\right).$$ (2.2)

Thus, the alternative frame equations is obtaind as:

$$\left(\begin{array}{c} j_{1}{\;}^{\prime }\\ j_{2}{\;}^{\prime }\\ j_{3}{\;}^{\prime } \end{array}\right)=\left(\begin{array}{ccc} \;\;0 & \kappa_{1} & \kappa_{2}\\ -\kappa_{1} & \;0 & \;0\\ -\kappa_{2} & \;0 & \;0 \end{array}\right)\left(\begin{array}{c} j_{1}\\ j_{2}\\ j_{3} \end{array}\right)=\varpi \times \left(\begin{array}{c} j_{1}\\ j_{2}\\ j_{3} \end{array}\right).$$ (2.3)

Here, $\varpi =\kappa (s)e_{3}$ represents the angular velocity vector of the BF. It follows that:

$$\begin{array}{c} \kappa_{1}(u)=\kappa \cos\vartheta \text{, }\kappa_{2}(u)=\kappa \sin\vartheta \text{, }\\ \vartheta (u)=\tan^{-1}\left(\frac{\kappa_{2}}{\kappa_{1}}\right);\;\kappa_{1}\neq 0,\\ \vartheta (u)-\vartheta_{0}=-\underset{s_{0}}{\overset{s}{\int }}\tau (u)du. \end{array}\}$$ (2.4)

By comparing Eq (2.1) with Eq (2.3), the relative velocity satisfies:

$$\omega (u)-\omega (u)=\tau (u)e_{1}(u).$$ (2.5)

This result indicates that the SFF incorporates an additional rotation around the tangent, with a speed equal to the torsion τ(u). Consequently, the integral formula in Eq (2.4) provides a method for computing the BF by eliminating the undesired rotation of the SFF. Notably, the SFF and BF coincide for planar curves where τ(u)=0, aligning with Klok’s findings [23].

**Lemma 2.1.** Let γ(u) be a unit speed curve with nonvanishing curvature κ(u) and torsion τ(u) . The natural mate of γ(u) is defined as the unit speed curve α(u) whose tangent vector is given by $𝐭(u)=\overset{u}{\underset{0}{\int }}e_{2}(u)du$. The pair {γ(u), α(u)} is referred to as the natural couple [17].

For the SFF of of the axial curve α(u), differentiating the tangent vector 𝐭(u) with respect to *u* gives


$$t{\;}^{\prime }(u)=e_{2}{\;}^{\prime }(u)=-\kappa e_{1}+\tau e_{3}.$$

Hence, the curvature of α(u) is


$$\kappa_{\alpha }(u)=\left\|e_{2}{\;}^{\prime }(u)\right\|=\sqrt{\kappa^{2}+\tau^{2}}.$$

The corresponding unit principal normal vector is therefore


$$p(u)=\frac{t{\;}^{\prime }(u)}{\kappa_{\alpha }(u)}=\frac{-\kappa e_{1}+\tau e_{3}}{\sqrt{\kappa^{2}+\tau^{2}}}.$$

The unit binormal vector is obtained as


$$b(u)={t\times p=e}_{2}\times \frac{-\kappa e_{1}+\tau e_{3}}{\sqrt{\kappa^{2}+\tau^{2}}}=\frac{\tau e_{1}+\kappa e_{3}}{\sqrt{\kappa^{2}+\tau^{2}}}.$$

In matrix form, the relationship between the frames $\left\{e_{1}(u\right)$, $e_{2}(u)$, $e_{3}(u)$} and {t(u), p(u), b(u) can be expressed as

$$\left(\begin{array}{c} t\\ p\\ 𝐛 \end{array}\right)=\left(\begin{array}{ccc} 0 & 1 & 0\\ -\frac{\kappa }{\sqrt{\kappa^{2}+\tau^{2}}} & 0 & \frac{\tau }{\sqrt{\kappa^{2}+\tau^{2}}}\\ \frac{\tau }{\sqrt{\kappa^{2}+\tau^{2}}} & 0 & \frac{\kappa }{\sqrt{\kappa^{2}+\tau^{2}}} \end{array}\right)\left(\begin{array}{c} e_{1}\\ e_{2}\\ e_{3} \end{array}\right).$$ (2.6)

This transformation establishes the local correspondence between the SFF {$e_{1}(u)$, $e_{2}(u)$, $e_{3}(u)$} and the secondary frame {t(u), p(u), b(u) associated with the curve α(u). The frame {t(u), p(u), b(u) will serve as a foundational reference in the subsequent construction and differential analysis of the swept surface.

A surface ℳ is defined by

$$ℳ:y(u,v)=\left(y_{1}\left(u,v\right),y_{2}\left(u,v\right),y_{3}\left(u,v\right)\right),\;\;(u,v)\in 𝔻\subseteq {\mathbb{R}}^{2}.$$ (2.7)

If $y_{j}(u,v)=\frac{\partial y}{\partial j}$, the surface normal vector is given by

$$n(u,v)=\frac{y_{u}\times y_{v}}{\left\|y_{u}\times y_{v}\right\|}.$$ (2.8)

The 1st fundamental form is

$$ℐ={𝔩}_{11}du^{2}+2{𝔩}_{12}dudv+{𝔩}_{22}dv^{2},$$ (2.9)

where ${𝔩}_{11}=<y_{u},y_{u}>$, ${𝔩}_{12}=<y_{u},y_{v}>,\;{𝔩}_{22}=<y_{v},y_{v}>$. The second fundamental form is

$$II={𝔥}_{11}du^{2}+2{𝔥}_{12}dudv+{𝔥}_{22}dv^{2},$$ (2.10)

where ${𝔥}_{11}=<y_{uu},n>$, ${𝔥}_{12}=<y_{tu},n>,\;{𝔥}_{22}=<y_{vv},n>$.

A ruled surface in ${𝔼}^{3}$ is represented as

y(u,v)=α(u)+v𝐞(u), v∈ℝ, (2.11)

where α(u) is known as the base (directrix) curve, and ‖𝐞‖=1. The lines $\alpha (u_{0})+v𝐞(u_{0})$ are called rulings. A developable surface is characterized by a constant surface normal along each ruling. The surface y(u,v) is developable if and only if

$$\det(\alpha {\;}^{\prime },e,e{\;}^{\prime })=0.$$ (2.12)

For clarification, in the present work, the natural mate curve is defined in an integral form, where the tangent direction of the mate is obtained through the accumulated transport of the principal normal along the generating curve. This formulation is fully consistent with the classical definition introduced by Sharief et al. [17], in which the tangent of the mate coincides locally with the principal normal of the original curve. The adopted integral representation therefore does not alter the geometric meaning but rather generalizes it, providing a global realization of the mate curve that remains equivalent to the standard definition under regular conditions.

## 3 Main results

In this section, it is illustrated an instance of a swept surface constructed using the natural mate of a spatial unit speed curve. In particular, the swept surface corresponding to α(u) is generated as the envelope of a one parameter family of unit spheres whose centers lie along α(u). Notice that the points of tangency between these spheres and the surface form great circles on each sphere. These circles are contained within the plane spanned by $Sp\{e_{1}$, $e_{3}\}$ at every point along α(u). Denote by y the mapping that links each point on the surface to its corresponding point on the axial curve. With this setup, the surface ℳ is defined as follows:


ℳ:y=α(u)+r.

The profile curve for the swept surface is defined by


$$r(v)=f(v)e_{1}(u)+g(v)e_{3}(u),\;u\in \mathbb{R},$$

with the condition $f^{2}(v)$  +  $g^{2}(v)=1$, which guarantees that r(v) describes a unit circle in the rectifying plane of the axial curve α(u). This profile curve may be constructed in either two or three dimensions, and it is assumed to intersect the axial curve α(u) during the formation of the surface. By combining these elements, the parametric representation of the swept surface becomes

$$ℳ:y(u,v)=\alpha (u)+f(v)e_{1}(u)+g(v)e_{3}(u).$$ (3.1)

**Remark 1**. Notice that if α(u) is a circle, the resulting swept surface forms a torus. Conversely, when α(u) is a straight line, the swept surface becomes a circular cylinder, with α(u) as symmetry axis.

Next, it is examined how the differentiability of the profile curve r(v) relates to the surface ℳ. For convenience, assume that ${\overset{.}{f}}^{2}+{\overset{.}{g}}^{2}=1$; ($\frac{d}{dv}$). The SFF of r(v) is given by its tangent vector

$$t_{r}(v)=\overset{.}{f}(v)e_{1}(u)+\overset{.}{g}(v)e_{3}(u),$$ (3.2)

and its associated principal normal, defined via the cross product with $e_{2}$,

$$p_{r}(v)=e_{2}\times t_{p}=\overset{.}{g}(u)e_{1}-\overset{.}{f}(u)e_{3}.$$ (3.3)

Furthermore, the tangent vectors of y(u,v) are determined by

$$\begin{array}{c} y_{u}(u,v)=(1+\kappa f-\tau g)e_{2},\\ y_{v}(u,v)=\overset{.}{f}e_{1}+\overset{.}{g}e_{3}. \end{array}\}$$ (3.4)

From these, the unit normal vector is computed as

$$n(u,v):=\frac{y_{u}\times y_{v}}{\left\|y_{u}\times y_{v}\right\|}=\overset{.}{g}(u)e_{1}-\overset{.}{f}(u)e_{3}.$$ (3.5)

This result confirms that n(u,v) lies within the rectifying plane of the axial curve α(u). Moreover, since $<n,e_{2}>=0$, it is concluded that $n∥p_{r}$; in other words, they share the same orientation.

**Proposition 3.1**. For any point x located in the rectifying plane of the axial curve α(u), the derivative $x_{u}$ ( i.e., the tangent at x) is always parallel to $e_{2}$.

Based on Eqs (3.4), we obtain

$${𝔩}_{11}=(1+\kappa f-\tau g{)}^{2},\;{𝔩}_{12}=0,\;{𝔩}_{22}=1.$$ (3.6)

Furthermore, the second derivatives of **y** are given by

$$\begin{array}{c} {𝐲}_{uu}=-(1+f_{2}\kappa -g\tau )\left(\kappa e_{1}+\tau e_{3}\right)+\left(\kappa {\;}^{\prime }f-\tau {\;}^{\prime }g\right)e_{2},\\ {𝐲}_{vu}=(\kappa \overset{.}{f}-\tau \overset{.}{g})e_{2},\\ {𝐲}_{vv}=\overset{..}{f}e_{1}+\overset{..}{g}e_{3}. \end{array}\}$$ (3.7)

Thus, the coefficients of the 2nd fundamental metric become

$$\begin{array}{c} {𝔥}_{11}=(1+f\kappa -g\tau )(\tau \overset{.}{f}+\kappa \overset{.}{g}),\\ {𝔥}_{12}=0,\;{𝔥}_{22}=-\overset{.}{g}\overset{..}{f}+\overset{.}{f}\overset{..}{g}. \end{array}\}$$ (3.8)

Eqs (3.6) and (3.8) indicate that the coordinate net forms curvature lines (since ${𝔩}_{12}={𝔥}_{12}=0$). This means that the profile curve r(v) a planar geodesic and a curvature line. In CAGD, surfaces where the net consists of curvature lines are particularly valuable [2,6].

Regarding swept surfaces, one defines the offset surface by

$$q_{c}(u,v)=q(u,v)+cn(u,v),$$ (3.9)

which represents the offset of q(u,v) by a constant distance *c*. Then the offset surface $q_{c}(u,v)$ remains a swept surface, generated by the same axial curve α(u) and the offset profile curve $r_{c}(v)$.

From this stage of the analysis, it becomes evident that the constructed surface possesses an intrinsically organized geometry: its coordinate network forms an orthogonal system in which each family of parameter curves follows the principal directions of curvature. This implies that the generating profile behaves both as a planar geodesic and as a principal curve, providing a natural adaptation between the surface parametrization and its curvature structure. When the surface is offset by translating each point along the normal direction by a constant distance, this geometric harmony is preserved. The offset surface inherits the same configuration of curvature lines and retains the differential geometric integrity of the original surface, demonstrating that the process of normal displacement does not distort the intrinsic geometry but just translates it in space.

**Proposition 3.2**. Consider the swept surface defined in Eq (3.1). Let $r_{c}(v)$ denote the planar offset of the profile curve r(v) by a constant distance *c*. Then the resulting offset surface defined by Eq (3.9) remains a swept surface, generated by the same axial curve α(u) and the offset profile $r_{c}(v)$.

This proposition clarifies that offsetting the generating curve by a constant distance does not alter the geometric nature of the swept surface. The resulting shape remains within the same family of swept surfaces, preserving the original axial curve and its spatial motion. Mainly, the offset operation shifts the generating profile while maintaining the same geometric framework and continuity of the surface structure.

**Corollary 3.1**. Let ℳ be the swept surface given in Eq (3.1). Then:

1) The *u*-curve is a geodesic if and only if


$$\overset{.}{f}\kappa -\overset{.}{g}\tau =0,\text{ and }f\kappa {\;}^{\prime }-g\tau {\;}^{\prime }=0.$$

2) The *u*-curve is an asymptotic curve if and only if


$$\overset{.}{f}\tau +\overset{.}{g}\kappa =0.$$

**Proof**.

1) Form Eqs (3.5), and (3.7), the *u*-curve is a geodesic if and only if ${𝐪}_{uu}\times n=0$, which yields


$$\begin{array}{c} -\overset{.}{f}\left(\kappa {\;}^{\prime }r_{2}-\tau {\;}^{\prime }r_{3}\right)e_{1}-(\overset{.}{f}\kappa -\overset{.}{g}\tau )(1+f\kappa -g\tau )e_{2}\\ -\overset{.}{g}\left(\kappa {\;}^{\prime }f-\tau {\;}^{\prime }g\right)e_{3}=0. \end{array}\}$$

Since $e_{1}$, $e_{2}$ and $e_{3}$ are linearly independent unit vectors, the coefficients must vanish, leading to the stated conditions.

2) Similarly, the *u*-curve is an asymptotic curve if and only if $<n,q_{uu}>=0$. This condition simplifies to


$$(\overset{.}{f}\tau +\overset{.}{g}\kappa )(1+f\kappa -g\tau )=0.$$

Since 1+fκ−gτ≠0 (as given by Eq (3.4)), we deduce that $\overset{.}{f}\tau +\overset{.}{g}\kappa =0$, which completes the proof. ∎

The corollary explains that the geometric behavior of the *u*-curve depends on how its acceleration aligns with the surface geometry. When the curve’s motion remains entirely within the tangent plane, it traces the path of least curvature, hence it becomes a geodesic. Conversely, when its bending direction coincides with the surface’s tangent plane rather than pointing outward, the curve follows an asymptotic path. In both cases, the relationships among the curvature and torsion functions govern whether the curve bends within or away from the surface, linking the local differential geometry of the generating motion to the intrinsic geometry of the swept surface.

### 3.1 Singularity and convexity

Singularity and convexity play fundamental roles in determining the geometric behavior of swept surfaces. Their influence on the structure of the surface ℳ is examined in detail below. The surface M exhibits singularities precisely when

$$\left\|y_{u}\times y_{v}\right\|=1+f\kappa -g\tau =0.$$ (3.10)

From this condition, together with the profile curve constraint *f*^2^  +  *g*^2^ = 1, singularities occur under the following setting. Let $r:=\sqrt{\kappa^{2}+\tau^{2}}$and assume that real solutions exist (as discussed below). Writing (f,g)=(cosϑ,sinϑ), we obtain


κcosϑ−τsinϑ=rcos(ϑ+φ)=−1,

where cosφ=κ/r, sinφ=τ/r. Hence


$$\cos(\vartheta +\varphi )=-\frac{1}{r}.$$

For real solutions to exist, we require r≥1 (i.e. $\kappa^{2}+\tau^{2}\geq 1$). Let $a:=\sqrt{r^{2}-1}=\sqrt{\kappa^{2}+\tau^{2}-1}$. Then, the two solutions for (*f*,*g*) are given by

$$f(u)=\frac{-\kappa \pm \tau a}{r^{2}}\text{, }g(u)=\frac{\tau \pm \kappa a}{r^{2}}\text{,}$$ (3.11)

where the same choice of sign is taken in both numerator entries. These relations remain valid in the limiting or special cases; for instance, when τ=0 one obtains

$$f(u)=\frac{-1}{\kappa }\text{, }g(u)=\pm \frac{1}{\kappa }\sqrt{\kappa^{2}-1}\text{.}$$ (3.12)

Substituting these expressions from Eqs (3.11) into Eq (3.1), the singular curve ofℳ can be expressed as

$${𝐜}_{\pm }(u)=\alpha (u)+\frac{1}{r^{2}}[(-\kappa \pm \tau a)e_{1}(u)+(\tau \pm \kappa a)e_{3}(u)].$$ (3.13)

A crucial geometric insight is that singular points appear when the profile curve r(u) intersects the instantaneous axis of rotation (ISA), defined by

ℒ(u)={(f(u),g(u)∣1+fκ−gτ=0. (3.14)

Geometrically, the singularity condition corresponds to the instant when the axial motion coincides with the generating profile, producing a local breakdown of immersion manifested as a fold or cusp on the envelope. Beyond its formal derivation, this phenomenon describes the geometric synchronization between the spine motion and the generating circle, which provides an intuitive physical picture of singularity formation. Likewise, the developable configurations cylindrical, conical, and tangential reflect pure bending states in thin shell mechanics, where stresses are distributed along generators without stretching, and also predict stable optical reflection patterns and minimal energy configurations. These interpretations suggest that the derived geometric conditions are not only theoretical but also predictive in engineering contexts such as robotic path planning, fluid surface modeling, and the stability analysis of thin walled structures.

Based on this, we derive the following corollary:

**Corollary 3.2.** Let ℳ be a swept surface as defined in Eq (3.1), where both the axial curve α(u) and the profile curve r(u) possess nonzero curvature. Then M entirely regular provided that

1+fκ−gτ≠0, (3.15)

for all values of *u*, and *v*.

#### 3.1.1 Convexity and parabolic curves

Convex surfaces are fundamental in geometry and optimization, playing a key role in stability and minimization properties. In diverse applications, such as sculptured surface design and layered manufacturing, understanding the conditions that ensure convexity or lead to parabolic points on a surface is essential [9–11]. Consequently, we analyze the criteria under which parametric nets generate parabolic curves as follows: For the swept surface described by Eq (3.1), its convexity depends on its geometric characteristics. Assuming ${𝔩}_{12}={𝔥}_{12}=0$, and utilizing Eqs (3.6) and (3.8), the Gaussian curvature is expressed as:

$$𝒦(u,v)=\frac{{𝔥}_{11}{𝔥}_{22}-{𝔥}_{12}^{2}}{{𝔩}_{11}{𝔩}_{22}-{𝔩}_{12}^{2}}=-\frac{\left(\tau \overset{.}{f}+\kappa \overset{.}{g})(-\overset{.}{g}\overset{..}{f}+\overset{.}{f}\overset{..}{g}\right)}{1+\kappa f-\tau g}.$$ (3.16)

Furthermore, the curvature of the isoparametric *v*-curves, where *u* remains constant, is given by:

$$r_{1}(u_{0},v)=\frac{\left\|\overset{.}{r}\times \overset{..}{r}\right\|}{{\left\|\overset{.}{r}\right\|}^{2}}=-\overset{.}{g}\overset{..}{f}+\overset{.}{f}\overset{..}{g}.$$ (3.17)

Similarly, the curvature of the isoparametric *u*-curves, where *v* is constant, is:

$$r_{2}(u,v_{0})=\frac{\left\|y_{u}\times y_{v}\right\|}{{\left\|y_{u}\right\|}^{2}}=\frac{1}{1+\kappa f-\tau g}.$$ (3.18)

Using Eqs (2.2) and (3.5), the normal vector n(u,v) is expressed as:

n(u,v)=cosϕp+sinϕb, (3.19)

where

$$\cos\phi =\frac{\tau \overset{.}{f}+\kappa \overset{.}{g}}{\sqrt{\kappa^{2}+\tau^{2}}}\text{ and }\sin\phi =\frac{-\kappa \overset{.}{f}+\tau \overset{.}{g}}{\sqrt{\kappa^{2}+\tau^{2}}}.$$ (3.20)

Substituting Eqs (3.16)–(3.19) into Eq (3.15), the Gaussian curvature reduces to:

$$𝒦(u,v)=-r_{1}(u_{0},v)r_{2}(u,v_{0})\cos\phi .$$ (3.21)

To characterize the structure of ℳ, we aim to identify the curves formed by parabolic points, i.e., points where 𝒦(u,v)=0. These curves distinguish between the hyperbolic regions (𝒦<0, indicating non-convexity), and elliptic regions (𝒦>0, signifying local convexity) partitions of ℳ. From Eq (3.20), three key conditions emerge when 𝒦=0 are:

Case (1) arises when $r_{1}(u_{0},v)=0$, meaning the profile curve r(v) simplifies to a straight line. From Eq (3.16), this implies

$$r_{1}(u_{0},v)=0=\left\|\overset{.}{r}\times \overset{..}{r}\right\|=0\Leftrightarrow \overset{.}{r}\times \overset{..}{r}=0\Leftrightarrow \overset{.}{r}∥\overset{..}{r},$$ (3.22)

indicating that a flat segment or an inflection point in r(v) generates a parabolic curve along *u* = const. in certain regions of M.

Case (2) occurs when $r_{2}(u,v_{0})=0$. If $r_{2}(u,v_{0})=0$, then the axial curve α(u) degenerates into a straight line. Similarly, a flat segment or an inflection point in α(u) results in a parabolic curve along v=const. on portions of ℳ.

Case (3) happens when ϕ=π/2. From Eqs (3.18) and (3.19), we obtain the condition n(u,v)∥b, which leads to

$$\tau \overset{.}{f}+\kappa \overset{.}{g}=0\text{ and }-\kappa \overset{.}{f}+\tau \overset{.}{g}=\sqrt{\kappa^{2}+\tau^{2}}.$$ (3.23)

This implies that the axial curve α(u) is not only a curvature line but also asymptotic.

**Corollary 3.3.** Consider a swept surface as defined in Eq (3.1), where both the natural mate curve and the profile curve maintain nonzero curvatures everywhere. The surface possesses parabolic points if and only if Eqs (3.20) hold for $(u,v)\in 𝔻\subseteq {\mathbb{R}}^{2}$.

**Example 3.1**. Consider the parametric representation of the curve:


γ(u)=(cosu,sinu,0), 0≤u≤2π.

For this curve, the structure of the SFF apparatus is given as follows:


$$e_{1}(u)=(-\sin u,\cos u,0)\text{, }e_{2}(q)=(-\cos u,-\sin u,0),\;$$

$$e_{3}(q)=(0,0,1),\;\kappa (u)=1,\;\tau (u)=0.$$

Applying Lemma 2.1, the associated natural mate curve (blue) of γ(u) is determined as:


α(u)=(−sinu,cosu,0).

**Fig 1:**
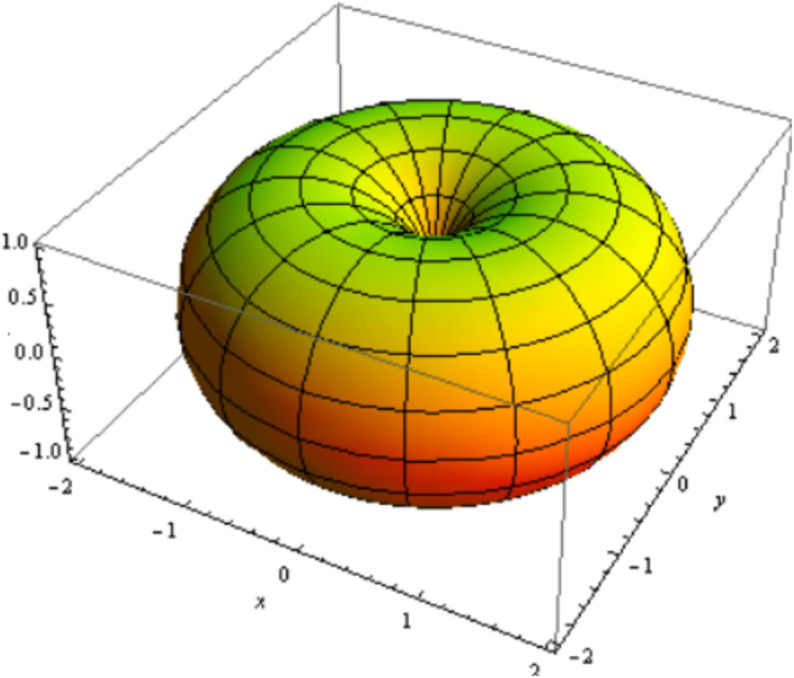
ℳ with its axial curve α(u).

From this, the singular curve is found to be 𝐜(u)=0, indicating that the singularity occurs only at the origin. Now, considering the profile curve defined by r(u)=(cosv,0,sinv), with 0≤u≤2π, we construct the swept surface ℳ as:


ℳ:y(u,v)=(−(1+cosv)sinu,(1+cosv)cosu,sinv),

for 0≤u, v≤2π, as depicted in Fig 1.

**Example 3.2.** Given the the unit-speed curve


$$\gamma (u)=(\sqrt{1-\sin u},\sqrt{1+\sin u},\frac{1}{\sqrt{2}}\cos^{-1}(\sin u)),\;0\leq u\leq 2\pi .$$

For this curve, the associated Serret Frenet frame vectors are given by


$$e_{1}(u)=(\frac{-\cos u}{2\sqrt{1-\sin u}},\frac{\cos u}{2\sqrt{1+\sin u}},\frac{-\cos u}{\sqrt{2-2\sin^{2}u}}),\;$$

$$e_{2}(u)=(\frac{\sqrt{1-\sin u}}{\sqrt{2}},-\frac{\sqrt{1+\sin u}}{\sqrt{2}},0),$$

$$e_{3}(u)=(\frac{-\cos u}{2\sqrt{1-\sin u}},\frac{\cos u}{2\sqrt{1+\sin u}},\frac{\cos u}{\sqrt{2-2\sin^{2}u}}).$$

where the curvature and torsion satisfy $\kappa (u)=-\tau (u)=\frac{1}{2\sqrt{2}}$. Applying Lemma 2.1, the natural mate (axial) curve corresponding to γ(u) is obtained as


$$\alpha (u)=(-\frac{\cos(\frac{\pi }{4}-\frac{u}{2})\sqrt{2-2\sin u}}{\cos(\frac{\pi }{4}+\frac{u}{2})},-\frac{\cos(\frac{\pi }{4}+\frac{u}{2})\sqrt{2+2\sin u}}{\cos(\frac{\pi }{4}-\frac{u}{2})},0).$$

From Eq (3.11), and noting that $\kappa (u)=-\tau (u)=\frac{1}{2\sqrt{2}}$, the expression under the square root in the auxiliary functions is


$$\kappa^{2}+\tau^{2}-1=-\frac{3}{4}\Rightarrow \sqrt{\kappa^{2}+\tau^{2}-1}=\frac{i\sqrt{3}}{2}.$$

Therefore, the functions *f*, and *g* take the form


$$f=\sqrt{2}(-1\pm \frac{i\sqrt{3}}{2})\text{, and }g=\sqrt{2}(-1\mp \frac{i\sqrt{3}}{2}).$$

**Fig 2:**
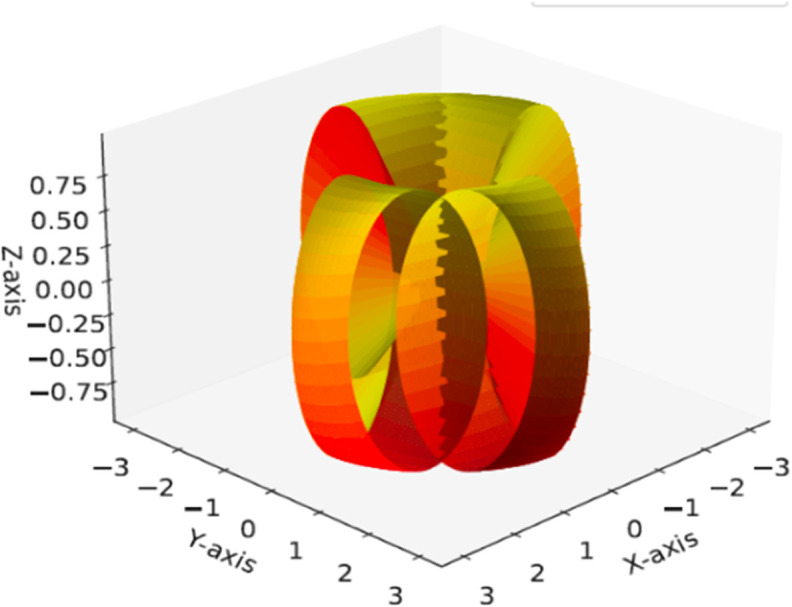
ℳ with its axial curve α(u) and striction curve 𝐜(u).

**Remark**: No real solutions exist since $\kappa^{2}$  +  $\tau^{2}<\frac{1}{4}$, implying that real striction curves do not occur on this surface. Geometrically, the striction curve corresponds to the locus of points on the ISA that remain closest to the trajectory α(u). When the combined curvature and torsion of the generating curve are too small, the ISA fail to intersect the surface envelope in real space. Consequently, the swept surface does not possess any real striction line—${ISA}^{\prime }s$ are skewed in such a way that their shortest distance points lie outside the real domain of ${𝔼}^{3}$. The resulting expressions


$${𝐜}_{\pm }(u)=\alpha (u)+4[(-1\pm \frac{i\sqrt{3}}{2})e_{1}(u)+(-1\mp \frac{i\sqrt{3}}{2})e_{3}(u)],$$

represent imaginary striction curves, corresponding to complex conjugate trajectories in the extended complex space. Thus, the corresponding swept surface pencil is given by


$$ℳ:q(u,v)=\alpha (u)+\cos ve_{1}(u)+\sin ve_{3}(u).$$

as illustrated in Fig 2, for 0≤u, v≤2π.

### 3.2 Developable surfaces

We now establish the conditions under which the swept surface is developable. Hence, we have the matter, that the profile curve is a line. Take $r(u)={\left(0,0,u\right)}^{T}$ for epitome, the corresponding developable surface constructed by α(𝔲) is as follows:

$$ℳ:𝔮(𝔲,𝔱)=\alpha (𝔲)+t{𝔢}_{3}(𝔲),\;𝔱\in \mathbb{R}.$$ (3.24)

Correspondingly, for r(u)=(u, $0,\;0){\;}^{T}$, the parameterization

$${ℳ}^{⊥}:{𝔮}^{⊥}(𝔲,𝔱)=\alpha (𝔲)+t{𝔢}_{1}(𝔲),\;𝔱\in \mathbb{R}.$$ (3.25)

is a developable surface. It is intelligible that 𝔮(𝔲,0)=α(𝔲) (${𝔮}^{⟂}(𝔲,0)=\alpha (𝔲)$), 0≤𝔲≤L, that is, the developable surface ${ℳ}^{⟂}$ (ℳ) interpolate the curve α(𝔲) as curvature line. Further, we possess

$$\frac{\partial 𝔮}{\partial 𝔲}\times \frac{\partial 𝔮}{\partial 𝔱}=\left(1-𝔱\tau \right){𝔢}_{1}(𝔲),$$ (3.26)

and

$$\frac{\partial {𝔮}^{⟂}}{\partial 𝔲}\times \frac{\partial {𝔮}^{⟂}}{\partial 𝔱}=-\left(1+t\kappa \right){𝔢}_{3}(𝔲).$$ (3.27)

Therefore, we possess ℳ (resp. ${ℳ}^{⊥}$) has no singular points iff 1−𝔱τ≠0 (resp. (1+𝔱κ≠0).

**Theorem 3.1**. Let ℳ be the swept surface marked by Eq (3.21). Then:

1) ℳ and ${ℳ}^{⊥}$ associated over α(u) at a right angle,

2) α(u) is a joint curvature line of ℳ and ${ℳ}^{⊥}$.

**Theorem 3.2**. (Existence and uniqueness). There exists a unique developable surface marked by Eq (3.21).

**Proof**. For the existence, We consider the developable surface defined by Eq (3.21). Furthermore, since ℳ is a ruled surface, we can record

$$\begin{array}{c} ℳ:𝔮(𝔲,𝔱)=\alpha (𝔲)+t𝔢(𝔲),\;𝔱\in \mathbb{R},\\ 𝔢(𝔲)=a(𝔲){𝔢}_{1}+b(𝔲){𝔢}_{2}+c(𝔲){𝔢}_{3},\\ {\left\|𝔢(𝔲)\right\|}^{2}=a^{2}+b^{2}+c^{2}=1,\;𝔢{\;}^{\prime }(𝔲)\neq 0. \end{array}\}$$ (3.28)

It is apparent that ℳ is a developable surface iff

$$\det(\alpha {\;}^{\prime },𝔢,𝔢{\;}^{\prime })=0\Leftrightarrow ca{\;}^{\prime }-ac{\;}^{\prime }+b\left(c\kappa -a\tau \right)=0.$$ (3.29)

Via Eq (3.23), we also meet

$$\left({𝔮}_{u}\times {𝔮}_{t}\right)\left(𝔲,𝔱\right)=\pm \lambda \left(𝔲,𝔱\right){𝔢}_{1},$$ (3.30)

where λ(𝔲,𝔱) is a smooth function. Further, the normal vector $\left({𝔮}_{u}\times {𝔮}_{t}\right)$ at the point (𝔲,0) is

$$\left({𝔮}_{u}\times {𝔮}_{t}\right)\left(𝔲,0\right)=-a{𝔢}_{3}+c{𝔢}_{1}.$$ (3.31)

From Eqs (3.27), and (3.28), the following can be acquired:

a=0, and c=±λ(𝔲,0), (3.32)

which follows from Eq (3.26) that bcκ=0, which leads to *cb* = 0, with κ(𝔲)≠0. If (𝔲,0) is a regular point (i.e., λ(𝔲,0)≠0 ), then c(𝔲)≠0, and *b* = 0. Therefore, the direction of 𝔢(𝔲) is identical with ${𝔢}_{3}(s)$. This signifies that uniqueness holds ∎.

In Theorem, 3.2 we not only illustrate the existence and uniqueness of the developable surface, but also grant the attachment character of the surface. This result has significant implications for practical applications, such as in surface modeling. For model, a pencil of cylindrical cutters, which is recognized by the locomotion of cylindrical cutter over α(𝔲), can be gotten as follows:

$$\overset{―}{ℳ}:\overset{―}{𝔮}(𝔲,𝔱)=𝔮(𝔲,𝔱)+\varrho {𝔢}_{1}(𝔲),$$ (3.33)

where ϱ is cylindrical cutter radius. This surface is an offset developable surface of the surface 𝔮(𝔲,𝔱) . The normal vector can be gained as

$$\overset{―}{𝔫}(𝔲,𝔱)=\frac{{\overset{―}{𝔮}}_{𝔲}\times {\overset{―}{𝔮}}_{𝔱}}{\left\|{\overset{―}{𝔮}}_{𝔲}\times {\overset{―}{q}}_{𝔱}\right\|}={𝔢}_{1}(𝔲).$$ (3.34)

The derivative of Eq (3.30) with respect to 𝔲 is

$${\overset{―}{𝔮}}_{𝔲}(𝔲,𝔱)=q_{𝔲}(𝔲,𝔱)+\omega (𝔲)\times (\varrho {𝔢}_{1}(𝔲)).$$ (3.35)

From Eq (3.32) it follows that the vector $<{\overset{―}{𝔮}}_{𝔲},{𝔢}_{1}>=0$. Therefore, the vector ${𝔢}_{1}$ is columnar to the tool axis. Consequently, the circumstance surface of the cylindrical cutter and the developable surface 𝔮(𝔲,𝔱) have the joint normal vector and the distance among these two surfaces is the cylindrical cutter radius ϱ.

**Proposition 3.3**. If the developable surface as in Eq (3.21) with α(𝔲) as a curvature line, and the circumstance surface of cylindrical cutter $\overset{―}{𝔮}(𝔲,𝔱)$ are regular, these two surfaces are offsets developable surfaces.

As it is will known, there are three sorts of developable surfaces, the choked curve can be designated into three forms alike. In what follows, we will inspect the link through α(𝔲) and its iso-parametric developable. The 1st case is when,

$${𝔢}_{3}\times {𝔢}_{3}{\;}^{\prime }=0\Leftrightarrow \tau {𝔢}_{1}=0.$$ (3.36)

In this time, ℳ is coined a cylinder. Since ${𝔢}_{1}\neq 0$, then ℳ is a cylinder iff τ(u)=0. This signifies that the curve α(𝔲) is a planar curve. By comparable, we possess:

$${𝔢}_{3}\times {𝔢}_{3}{\;}^{\prime }\neq 0.$$ (3.37)

Thus ℳ is a non-cylinder. Then, the 1st derivation $\alpha {\;}^{\prime }$ is

$$\alpha {\;}^{\prime }(𝔲)=c{\;}^{\prime }(𝔲)+\sigma (𝔲){𝔢}_{3}{\;}^{\prime }(𝔲)+\sigma {\;}^{\prime }(𝔲){𝔢}_{3}(𝔲),$$ (3.38)

where $𝐜{\;}^{\prime }$ is the 1st derivation of the striction curve, σ(𝔲) is a smooth function. So,

$$<{𝔢}_{3}\times {𝔢}_{3}{\;}^{\prime },𝐜{\;}^{\prime }>=0.$$ (3.39)

In like manner, there are two issues which rescue Eq (3.36), as addressed in the following: The 1st one is when $𝐜{\;}^{\prime }=0$. This admit that the striction curve turn into a point, and the developable surface becomes a cone; the striction point of a cone is commonly mention to as the vertex. Therefore, the surface ℳ is a cone iff there exists a stationary point **c** and a regular function σ(𝔲) such that $\sigma \tau =-1,\;\sigma {\;}^{\prime }=0$, which reveal that

$$\sigma =const.=\frac{1}{\tau }\Leftrightarrow \tau (𝔲)=\tau ({𝔲}_{0}).$$ (3.40)

The 2nd issue is when $\tau (s)\neq \tau (s_{0})$, then $𝐜{\;}^{\prime }\neq 0$. Since $<{𝔢}_{3}$ × ${𝔢}_{3}{\;}^{\prime },𝐜{\;}^{\prime }>=0$, $<{𝔢}_{3},{𝔢}_{3}{\;}^{\prime }>=0$, and the condition for c to be striction curve is alike to $<𝐜{\;}^{\prime }$,${𝔢}_{3}{\;}^{\prime }>=0$ we can realize $𝐜{\;}^{\prime }\|e_{3}$. This detects the tangent surface is collected of the tangents of α(𝔲).

### 3.3 Examples

In the following section, we analyze the structure of a developable surface for which the generating curve is a line of curvature.

**Example 3.3**. Given γ(𝔲) be


γ(𝔲)=(cos𝔲,sin𝔲,0), 0≤𝔲≤2π.

Then,


$${𝔢}_{1}(𝔲)=(-\sin 𝔲,\cos 𝔲,0),$$

$${𝔢}_{2}(𝔲)=(-\cos 𝔲,-\sin 𝔲,0),$$

$${𝔢}_{3}(𝔲)=(0,0,1),\;\kappa (u)=1,\;\tau (𝔲)=0\text{. }$$

By a similar execution as in Example 3.1, we possess


α(𝔲)=(−sin𝔲,cos𝔲,𝔞), 𝔞=const.

**Fig 3:**
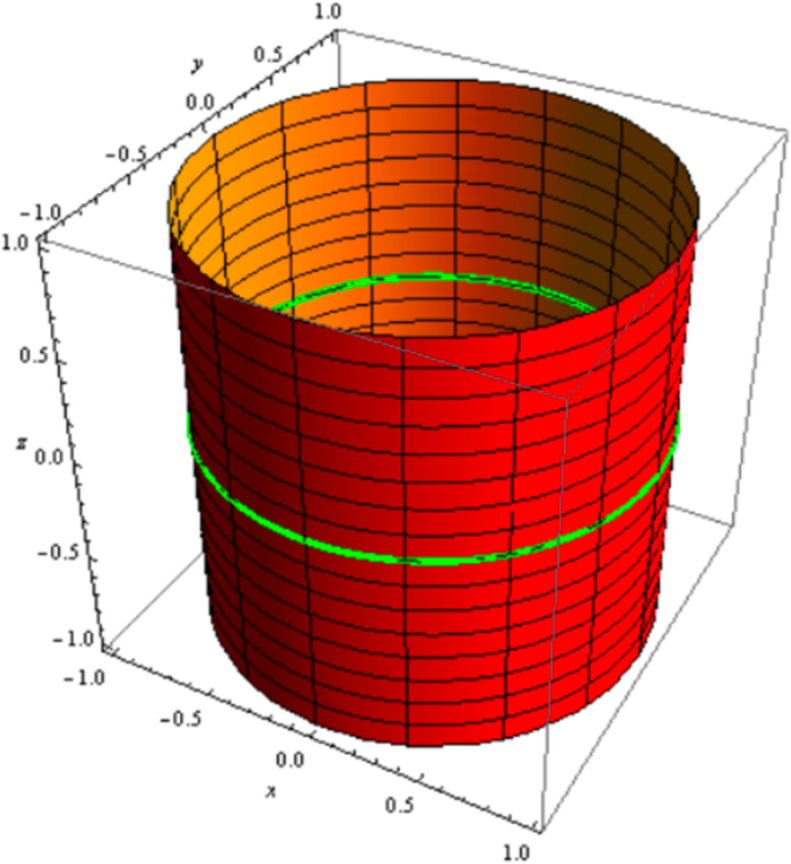
Cylinder with natural mate curve of a circle.

Then,


ℳ:𝔮(𝔲,𝔱)=(−sin𝔲,cos𝔲,𝔞+𝔱)

is a cylinder with natural mate curve of a circle as a curvature line; *a* = 0, and 0≤𝔱≤1 (Fig 3)

**Example 3.4.** Given the cylindrical helix


$$\gamma (𝔲)=\frac{1}{\sqrt{2}}(-\cos 𝔲,-\sin 𝔲,𝔲),\;\;0\leq 𝔲\leq 2\pi .$$

Then,


$$\begin{array}{c} {𝔢}_{1}(𝔲)=\frac{1}{\sqrt{2}}(\sin 𝔲,-\cos 𝔲,1),\\ {𝔢}_{2}(𝔲)=(\cos 𝔲,\sin 𝔲,0),\\ {𝔢}_{3}(𝔲)=\frac{1}{\sqrt{2}}(-\sin 𝔲,\cos 𝔲,1),\\ \alpha (𝔲)=\left(-\sin 𝔲,\cos 𝔲,a\right),\;a=con𝔲t.\text{,}\\ \kappa =\tau =\frac{1}{\sqrt{2}}\text{, with }\tau (𝔲)=\tau ({𝔲}_{0}). \end{array}\}$$

**Fig 4:**
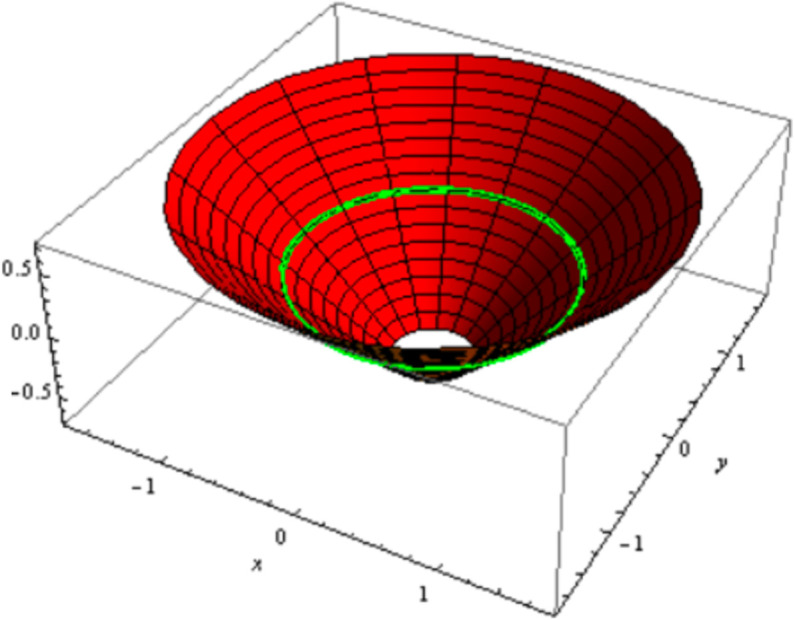
Cone with natural mate curve of a cylindrical helix.

Take *a* = 0 for specimen, then


$$ℳ:𝔮(𝔲,𝔱)=\left(-\sin 𝔲,\cos 𝔲,a\right)+t\frac{1}{\sqrt{2}}(-\sin 𝔲,\cos 𝔲,1),\;-1\leq 𝔱\leq 0.$$

is a cone surface with natural mate curve of a helix as a curvature line (See Fig 4); −1≤t≤0.

**Fig 5:**
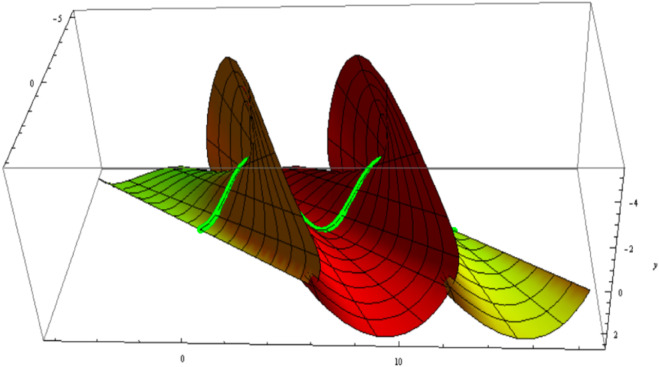
Tangential surface with natural mate curve of a slant helix.

**Example 3.5**. Let ℳ be a developable surface for which the base curve as in Example 3.1. If we set 𝔲=0, then τ(0)≠τ(𝔲), and


$$ℳ:𝔮(𝔲,𝔱)=\left(\frac{\sqrt{3}}{2}\sin2u+\frac{t}{4}\left(3\cos 𝔲-\cos3𝔲\right),\sqrt{3}\cos2u-\frac{t}{2}\sin2u,2u+\frac{\sqrt{3}}{2}\cos u\right),$$

is a tangential surface with natural mate curve of a slant helix as a curvature line; −1≤t≤0 (Fig 5).

## Conclusion

This study presents a geometric analysis of swept surfaces generated via natural mate curves. It establishes conditions under which these surfaces exhibit curvature lines, geodesics, singularities, and developable behavior. Singular points occur when the profile intersects the instantaneous rotation axis, and Gaussian curvature reveals parabolic curves separating convex and non-convex regions. Offset and developable variants including cylinders, cones, and tangents are rigorously characterized. The results offer valuable tools for applications in CAGD, manufacturing, and surface design. The originality of this work lies in presenting an explicit analytical formulation based on the Bishop frame for the generation of swept surfaces, ensuring geometric regularity even in the presence of torsion induced deformations an issue that conventional Frenet-based models have not efficiently addressed. This feature constitutes a significant advancement in computer-aided geometric design (CAD/CAM) applications, as it enables torsion continuity that yields smoother, collision free machining trajectories during offset surface generation. Moreover, the curvature adapted structure of the proposed surfaces provides a rigorous mathematical foundation for robotic end-effector path planning and motion analysis over geometrically complex surfaces, thereby enhancing kinematic precision in highly curved three-dimensional environments. In addition, the proposed framework opens new avenues in advanced manufacturing and digital design by enabling the accurate generation of developable and offset surfaces, which are of fundamental importance in mold design, sheet-metal forming, and layer-by-layer planning in additive manufacturing. Thus, the scientific contribution of this study extends the practical utility of the Bishop frame in geometric modeling far beyond traditional representations of tangent, cylindrical, or conical surfaces [9,12,16]. There are diverse occasions for further work. The methodology exercised here can be applied to the swept surfaces in another spaces such as Lorentz-Minkowski space, isotropic space, and etc.
