# Correction: Geometric and singularities insights of swept surfaces via the Bishop frame in Euclidean 3-Space

**DOI:** 10.1371/journal.pone.0352655

**Published:** 2026-06-25

**Authors:** Fatemah Mofarreh, Rashad Abdel-Baky, Munirah Alsahli

There is an error in affiliation 1 for authors Fatemah Mofarreh and Munirah Alsahli. The correct affiliation 1 is: Mathematical Science Department, College of Science, Princess Nourah Bint Abdulrahman University, Riyadh, Saudi Arabia.
